# Metatarsal metastasis from clear cell renal cell carcinoma: a case report and literature review

**DOI:** 10.1186/s12894-020-00588-4

**Published:** 2020-02-24

**Authors:** Hongzeng Wu, Ruoqi Han, Qianqian Zhang, Yi Zhao, Helin Feng

**Affiliations:** 1 Department of Orthopedics, The Fourth Hospital of Hebei Medical University, 12 Health Road, Shijiazhuang, Hebei 050011 People’s Republic of China; 2 Department of Breast Surgery, The Fourth Hospital of Hebei Medical University, 12 Health Road, Shijiazhuang, Hebei 050011 People’s Republic of China; 3 Department of Gynecology, Hebei Medical University Second Affiliated Hospital, 215 Heping Road, Shijiazhuang, Hebei 050011 People’s Republic of China

**Keywords:** Metatarsal metastasis, Renal cell carcinoma (RCC), Foot bone

## Abstract

**Background:**

Bone metastasis is known to occur in some patients with cancer, usually in the spine, pelvis or ribs, and less than 0.01% of patients have metastases in the foot bone, so metatarsal metastasis is quite rare. The initial symptoms of osseous metastases are swelling, pain, or both.

**Case presentation:**

We report a 68-year-old man with solitary metatarsal metastasis 26 months after a diagnosis of renal clear cell carcinoma. The patient suffered intermittent swelling of his right foot and pain for one year due to trauma and was not treated. The doctor attributed the symptoms to trauma, administering massage therapy and a plaster cast to the patient at the local clinic. After reviewing the medical records, we found that this patient had a history of clear cell renal cell carcinoma. The patient underwent radiological examination and open biopsy of the first metatarsal bone of the right foot. These findings confirmed that the patient had a metatarsal metastasis from clear cell renal cell carcinoma. The patient subsequently underwent right foot amputation. No local recurrence or distant metastasis was found after a 6-month follow-up.

**Conclusion:**

Clinicians should be aware of a history of renal cell carcinoma (RCC) and fully understand the patient’s past medical history. When treating patients with clear cell renal cell carcinoma who have unresolving bony pain or swelling, clinicians should always keep in mind the possibility of bone metastasis of RCC.

## Background

Metastatic disease of the skeleton often occurs in patients with malignancy, with bone damage and pain as the main manifestations. Malignant tumours prone to bone metastasis are breast cancer, lung cancer, kidney cancer, and other common primary cancers, including prostate cancer [1]. The sites of bone metastases are frequently localized in the axial skeleton, such as the spine and pelvis. Metastatic diseases of the distal knee and elbow joints are unusual. Bone metastasis of the foot is rare, occurring in approximately 0.01% of all metastatic bone diseases and is usually a late manifestation of disseminated disease [2, 3]. We report metatarsal metastasis from clear cell renal cell carcinoma in a 68-year-old man and review the related literature.

## Case presentation

**Fig. 1:**
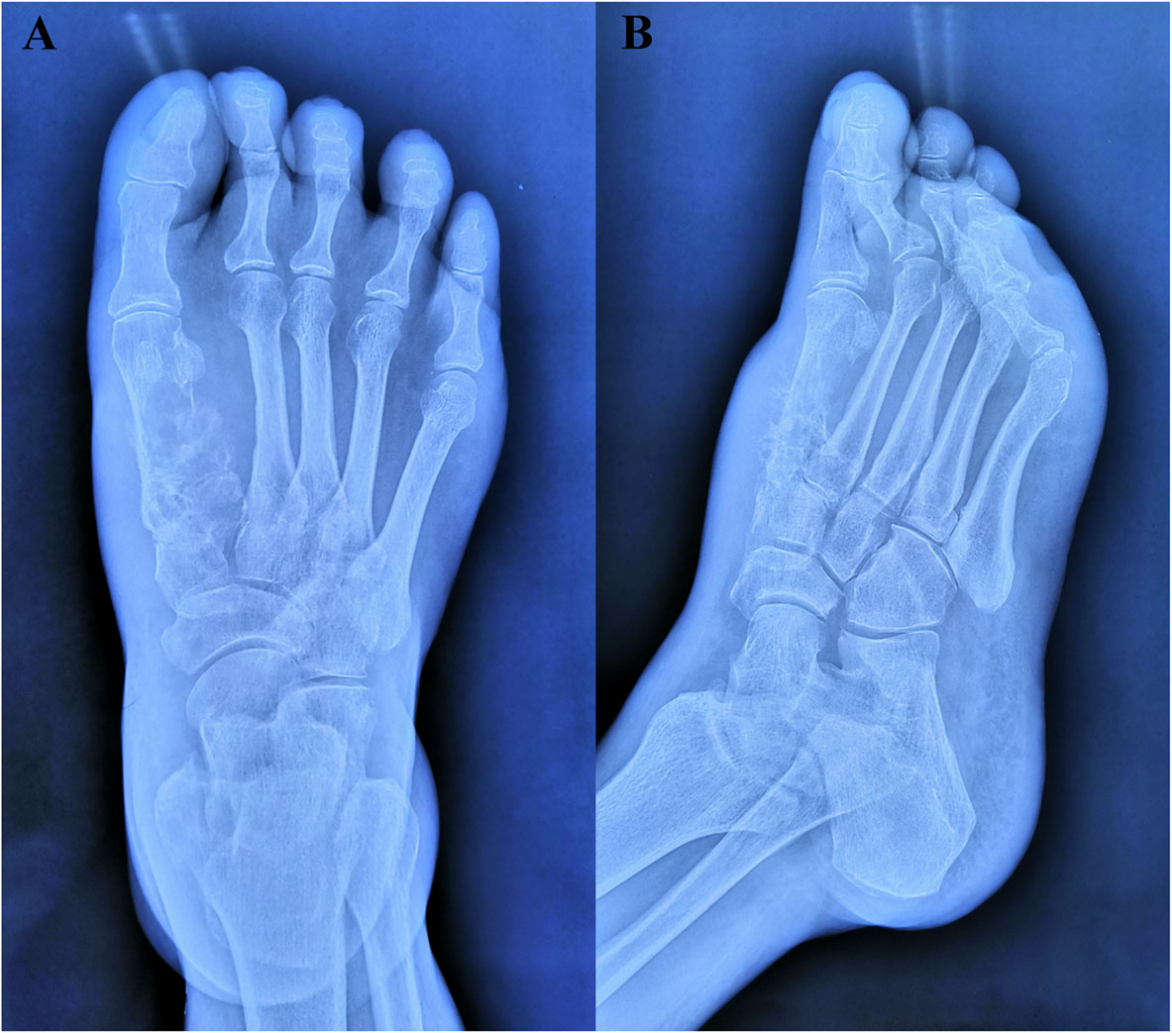
X-ray scan of the patient. **a** Posteroanterior radiograph. **b** Lateral radiograph

A 68-year-old male with intermittent swelling of his right foot and pain for one year was admitted in July 2019. The patient had a history of a mild right foot sprain that caused pain a year prior. Initially, the pain was relieved after rest but progressed with worsening pain and swelling after daily activities. At the local clinic, the doctor attributed the symptoms to trauma, administering massage therapy and a plaster cast to the patient, and the symptoms were alleviated. Two months before hospitalization, the patient’s right foot was continuously swollen with worsening, severe pain that limited ambulation. For further treatment, the patient came to our outpatient clinic and underwent X-ray examination of the right foot. The physical examination demonstrated that the soft tissue was mildly swollen. There was pain on palpation in the first metatarsus of the right foot. There was no paresthesia of the surrounding skin. The results of radiographs confirmed bone destruction of the first metatarsus of the right foot, which suggested underlying malignancy (Fig. 1a and b).

**Fig. 2:**
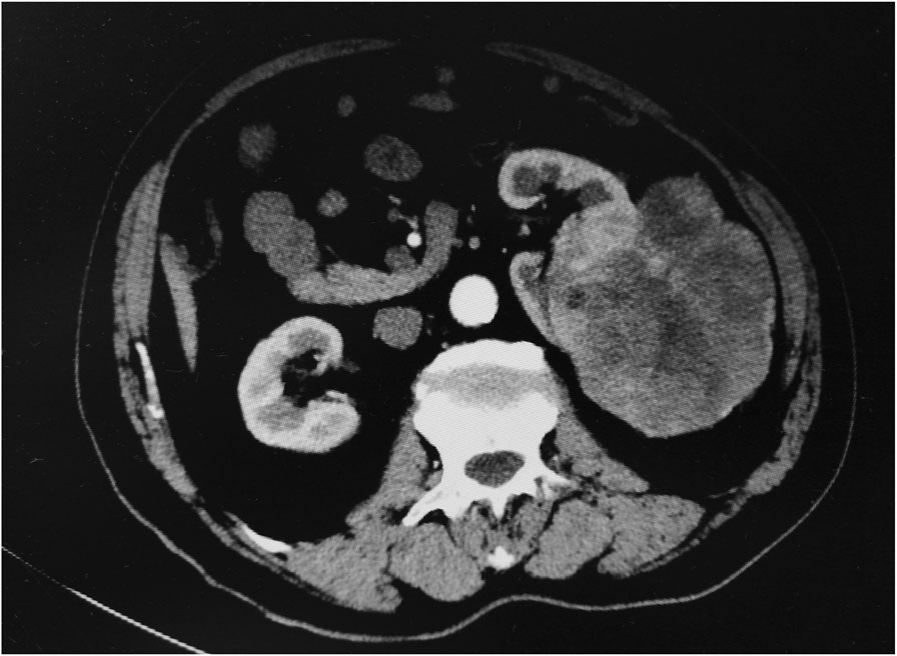
Abdominal CT showing a tumour in the left kidney

After reviewing the medical history, we discovered that the patient had a history of a large left renal mass discovered in April 2017 (Fig. 2). Computed tomography (CT) showed a round low-density shadow in the left inferior kidney, approximately 13*10 cm in size, with uneven density. There were flaky low-density shadows and point-like high-density shadows. Subsequently, the work-up was negative for metastatic disease, and radical nephrectomy was performed. Postoperative pathological results demonstrated stage II (T2N0M0) clear cell renal cell carcinoma (Fuhrman nuclear grade 2). After discharge, the patient was reviewed regularly in a polyclinic and received Chinese herbal treatment. Therefore, in the context of a known primary RCC, a metatarsal metastasis was suspected. The patient was admitted to the orthopaedic department for further treatment.

**Fig. 3:**
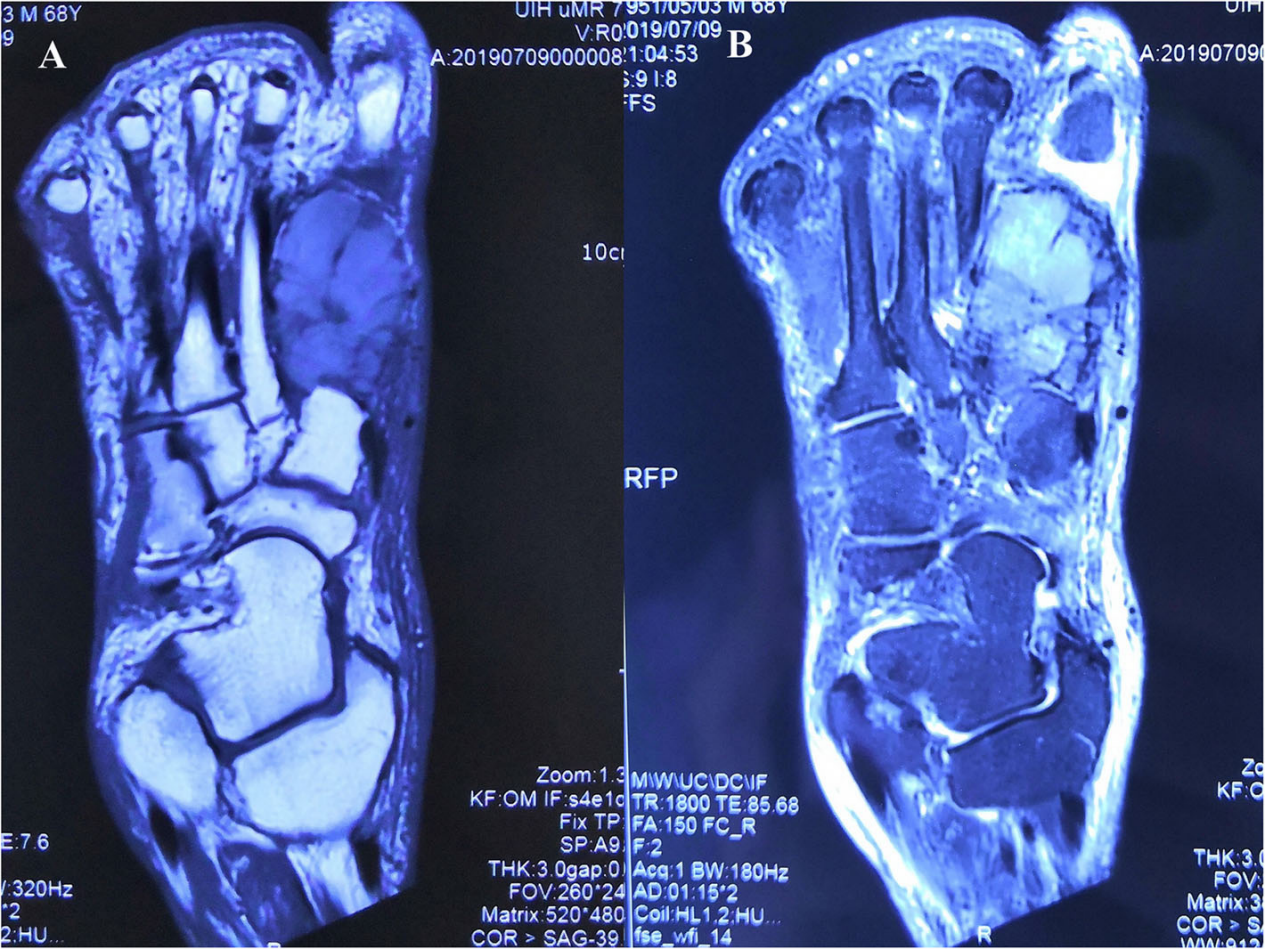
Foot magnetic resonance imaging scans of the patient. **a** T1-weighted images. **b** T2-weighted images

**Fig. 4:**
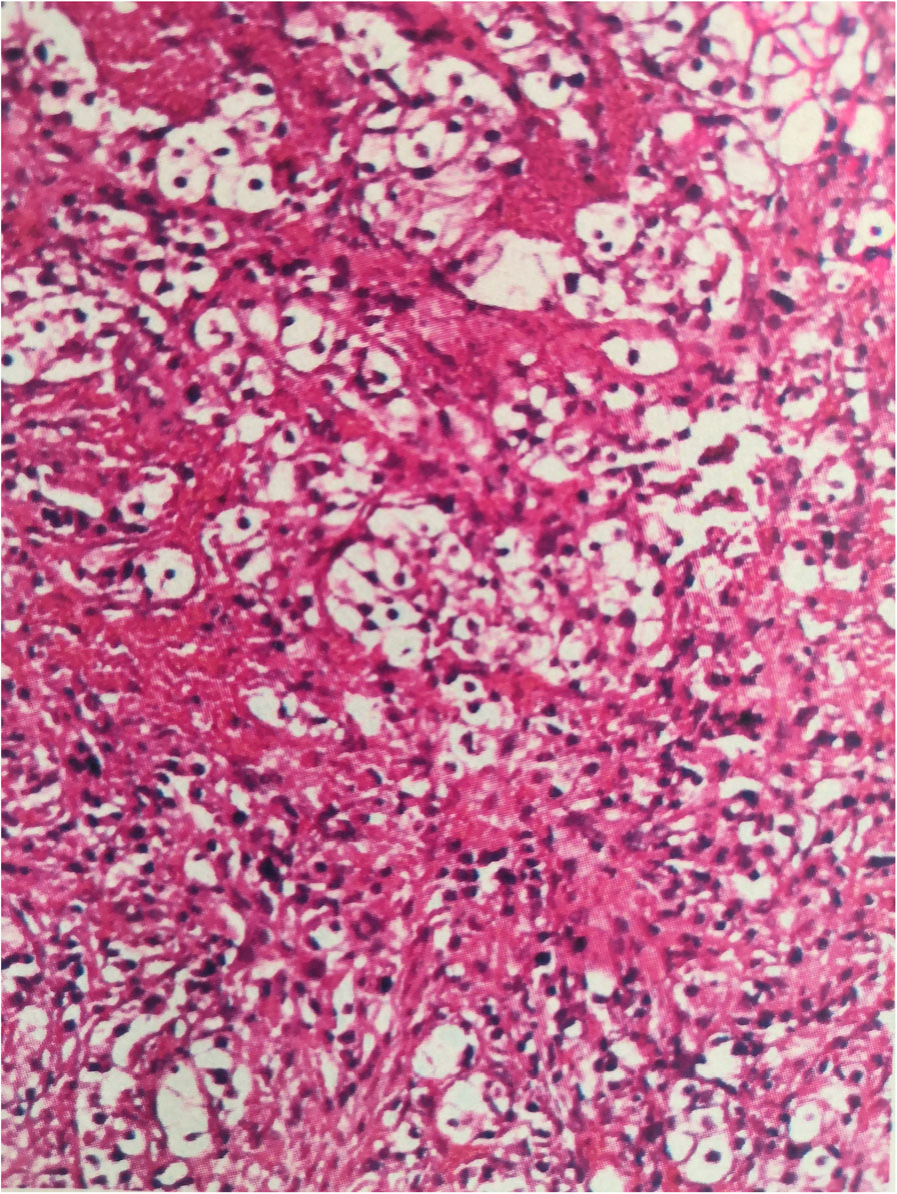
Postoperative pathology of the metatarsal bone

**Fig. 5:**
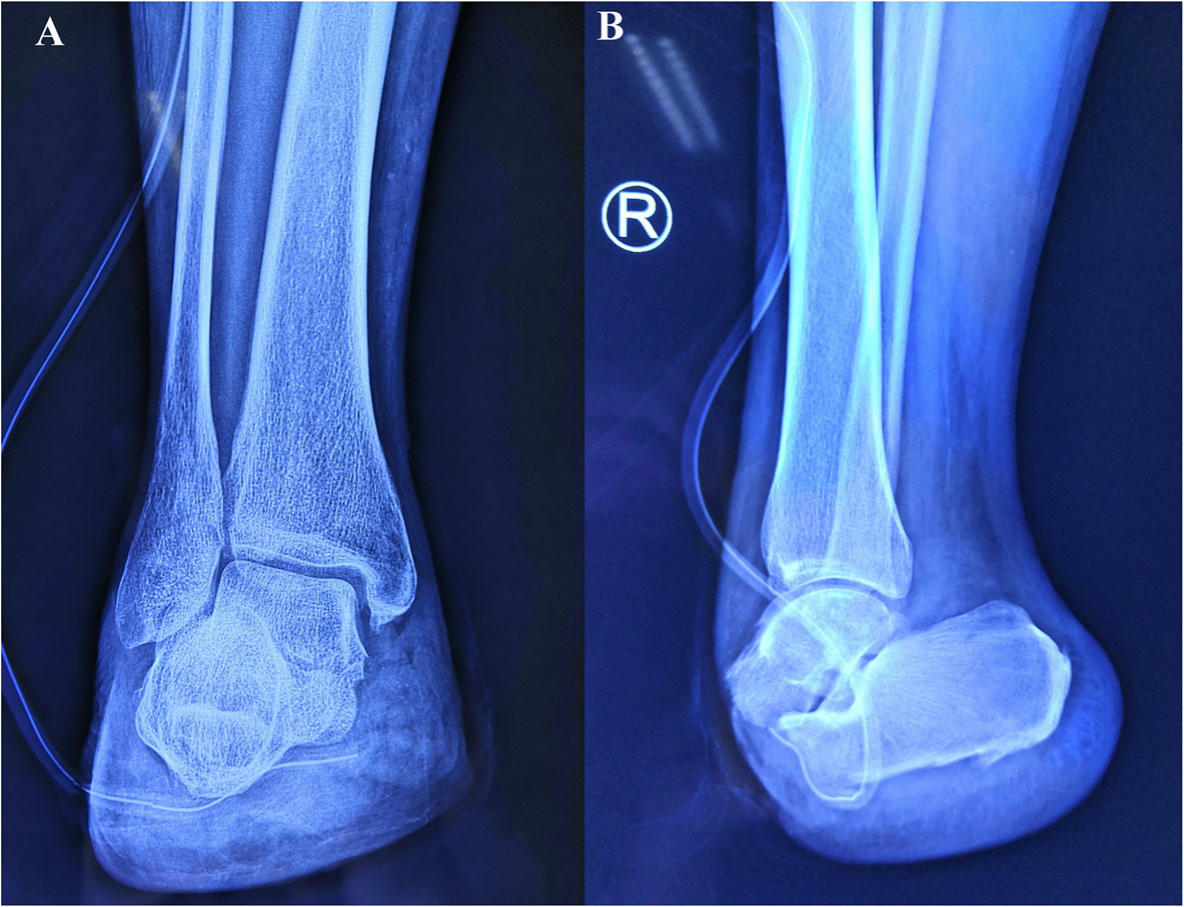
Postoperative X-ray scan of the patient. **a** Positive radiograph. **b** Lateral radiograph

After admission, the patient underwent magnetic resonance imaging (MRI), revealing a mass on the right first metatarsal bone with a less clear margin, approximately 5.19 cm in diameter, which had an isointense signal on T1-weighted images (Fig. 3a) and a mixed slightly high signal on T2-weighted images (Fig. 3b), with bone destruction, which was considered to be a malignant tumour. The results of the CT scan were consistent with those of MRI. After thorough examination and evaluation, no other distant metastatic lesions were found in the patient. Next, the patient underwent open biopsy of the first metatarsal bone of the right foot. Histological examination revealed clear cell carcinoma with nephrogenic implications (Fig. 4). Therefore, metatarsal metastasis from clear cell renal cell carcinoma was confirmed. The patient subsequently underwent right foot amputation (Fig. 5a and b). Six months after surgery, the patient walked with crutches and was re-examined at the orthopaedic clinic of our hospital. No local recurrence or distant metastasis was found.

## Discussion and conclusion

RCC is commonly known to metastasize to the lungs, bone, and brain, but RCC can metastasize to some atypical sites as well, such as skeletal muscle [4], scalp [5] and uvula [6], as described in the literature. This report describes the metastasis of clear cell renal cell carcinoma to the metatarsus. Previously reported malignant tumours that metastasized to the foot skeleton are listed in Tables 1 and 2. The most common primary cancer of foot bone metastasis is lung cancer, followed by renal and intestinal tumours [7, 50, 51]. In metastatic RCC, bone metastases occur in almost 30% of patients [52]. Bone metastases are a common site of relapse in many types of solid cancers. Most bone metastases occur in the spine, followed by the pelvis and long bones. Acrometastasis (defined as metastasis to the hand or foot) is quite rare [2]. The metastatic sites of foot bones are summarized in Table 3 according to previous case reports. The calcaneus is the foot bone most commonly involved [18, 35].

**Table 1:** Malignant tumors metastasized to the foot skeleton

Primary carcinoma	Number	(Refs.)
Lung cancer	11	[2, 7–16]
Cervical carcinoma	2	[16, 17]
Endometrial cancer	5	[18–20]
Prostatic cancer	5	[23–25]
Breast cancer	3	[3, 28, 29]
Intestinal tumor	7	[30, 31]
Bladder cancer	2	[3, 34]
Kidney cancer	7	[35–39]
Gastric adenocarcinoma	2	[42, 43]

**Table 2:** Malignant tumors metastasized to the foot skeleton

Others	Urothelial carcinoma of the urete [44], Submandibular gland carcinoma [45], Hypopharyngeal epidermoid carcinoma [9], Melanoma [46], Multiple myeloma [3],Humeral osteogenic sarcoma [30], Non-Hodgkin Lymphoma [47], Nasopharyngeal carcinoma [48], Esophagus carcinoma [49], Hypernephroma [30]

**Table 3:** Metastatic sites of foot bones

Location	Number	[Refs.]
Calcaneus	15	[3, 8, 16, 18, 22, 24, 26, 27, 29, 32, 38, 42]
Metatarsals	14	[2, 7, 10–12, 19, 31–35, 39, 46, 49]
Phalanges	13	[9, 17, 20, 21, 28, 36, 40, 41, 43, 45, 47, 48, 53]
Talus	1	[13]
Cuboid	4	[25, 32, 33, 47]
Navicular bones	3	[14, 23, 33]
Cuneiform bones	4	[2, 15, 33, 37]

In this case report, the clinician did not inquire about the patient’s previous medical history, nor did he perform any image examination for the foot for the patient upon first presentation. During the 1-year period, the patient developed intermittent swelling of the right foot with mild pain and an inability to walk without receiving definite diagnosis and timely treatment. Diagnostic delay may lead to pathological fractures; therefore, metastatic bone disease of the foot may affect the quality of life of patients, particularly as they are ambulating. However, the combination of medical history and imaging examination usually reveals the underlying diagnosis and provides a reference for treatment. For that reason, we report metatarsal metastasis from clear cell renal cell carcinoma in a 68-year-old man and review cases of foot bone metastasis of renal cancer reported in previous literature (Table 4). In these cases, the most common pathological type of foot bone metastasis was clear cell renal cell carcinoma, which represents the most common histology of renal carcinoma and has a worse prognosis than other RCCs [54–56]. All patients in Table 4 were male. Studies have reported a 4:1 male predominance in clear cell renal cell carcinoma with bone metastasis [57]. Almost all the patients received surgical treatment.

**Table 4:** Review of the previously reported cases of foot bone metastasis of renal cancer

Age	FS	Sex	Metastatic sites	Pathology	Treatment	[Refs.]
72	Y	Male	Phalanges	Clear cell carcinoma	Surgery	[36]
55	Y	Male	Metatarsals	Clear cell carcinoma	–	[35]
58	Y	Male	Cuneiform bones	Clear cell carcinoma	Surgery and Medication and Radiotherapy	[37]
59	N	Male	Calcaneus	Clear cell carcinoma	Surgery	[38]
–	–	–	Metatarsals	Adenocarcinoma	Surgery	[39]
55	Y	Male	Phalanges	Adenocarcinoma	Surgery	[40]
59	Y	Male	Phalanges	Clear cell carcinoma	Surgery	[41]

*FS* first presentation

As no therapeutic standard or guideline currently exists for RCC with bone metastasis. The median survival time of RCC with bone metastasis is usually below 24 months [57]. Acrometastases are often associated with extensive metastasis in other sites, so the prognosis is poor. The treatment is usually palliative and needs to be adapted to each patient’s individual condition. Relief of pain is often the therapeutic goal. Palliative treatment of bone metastases has always been conservative: radiotherapy, chemotherapy, immunotherapy, targeted therapy, bisphosphonates and analgesics [58]. More recently, minimally invasive techniques, including ethanol ablation, laser ablation, microwave ablation, cryoablation and radiofrequency ablation, have been used for painful bone lesions [59–63]. Surgical resection of bone metastasis from RCC has been reported to improve the prognosis of patients, and the effect is further improved for solitary metastasis if feasible [64]. These conditions were all met in our case. We believe that the best treatment for a single metastasis is always surgical excision if there is a long time from nephrectomy to the detection of isolated metastasis.

In conclusion, clinicians should fully understand the patient’s past medical history. Even though acrometastasis is rare, this diagnosis should be considered in any patient with a history of RCC, particularly male patients, with local pain and swelling. Appropriate clinical and radiographic evaluation of these patients is essential to offer timely local therapy that may improve prognosis and enhance patient quality of life.

## Data Availability

All data supporting the study are presented in the manuscript or available upon request.

## Abbreviations

CT: Computed Tomography
MRI: Magnetic Resonance Imaging
RCC: Renal Cell Carcinoma
